# Malaria Parasite Schizont Egress Antigen-1 Plays an Essential Role in Nuclear Segregation during Schizogony

**DOI:** 10.1128/mBio.03377-20

**Published:** 2021-03-09

**Authors:** Abigail J. Perrin, Claudine Bisson, Peter A. Faull, Matthew J. Renshaw, Rebecca A. Lees, Roland A. Fleck, Helen R. Saibil, Ambrosius P. Snijders, David A. Baker, Michael J. Blackman

**Affiliations:** a Malaria Biochemistry Laboratory, The Francis Crick Institute, London, United Kingdom; b Department of Biological Sciences, Institute of Structural & Molecular Biology, Birkbeck College, University of London, London, United Kingdom; c Centre for Ultrastructural Imaging, Guy’s Campus, King’s College London, London, United Kingdom; d Mass Spectrometry Proteomics Platform, The Francis Crick Institute, London, United Kingdom; e Advanced Light Microscopy, The Francis Crick Institute, London, United Kingdom; f Faculty of Infectious and Tropical Diseases, London School of Hygiene & Tropical Medicine, London, United Kingdom; Washington University School of Medicine

**Keywords:** SEA1, CENP-C, schizogony, egress, *Plasmodium falciparum*, malaria, SEA1

## Abstract

Malaria parasites cause disease through repeated cycles of intraerythrocytic proliferation. Within each cycle, several rounds of DNA replication produce multinucleated forms, called schizonts, that undergo segmentation to form daughter merozoites. Upon rupture of the infected cell, the merozoites egress to invade new erythrocytes and repeat the cycle. In human malarial infections, an antibody response specific for the Plasmodium falciparum protein PF3D7_1021800 was previously associated with protection against malaria, leading to an interest in PF3D7_1021800 as a candidate vaccine antigen. Antibodies to the protein were reported to inhibit egress, resulting in it being named schizont egress antigen-1 (SEA1). A separate study found that SEA1 undergoes phosphorylation in a manner dependent upon the parasite cGMP-dependent protein kinase PKG, which triggers egress. While these findings imply a role for SEA1 in merozoite egress, this protein has also been implicated in kinetochore function during schizont development. Therefore, the function of SEA1 remains unclear. Here, we show that P. falciparum SEA1 localizes in proximity to centromeres within dividing nuclei and that conditional disruption of SEA1 expression severely impacts the distribution of DNA and formation of merozoites during schizont development, with a proportion of SEA1-null merozoites completely lacking nuclei. SEA1-null schizonts rupture, albeit with low efficiency, suggesting that neither SEA1 function nor normal segmentation is a prerequisite for egress. We conclude that SEA1 does not play a direct mechanistic role in egress but instead acts upstream of egress as an essential regulator required to ensure the correct packaging of nuclei within merozoites.

## Abstract (precis)

Malaria is a deadly infectious disease. Rationally designed novel therapeutics will be essential for its control and eradication.

## INTRODUCTION

Plasmodium falciparum, the protozoan parasite responsible for the deadliest form of malaria, causes disease via repeated cycles of asexual growth within host red blood cells (RBCs). Merozoites invade RBCs and then grow within them, replicating their DNA over approximately 48 h to form multinucleate schizonts. The schizont cytoplasm is then divided into daughter cells, in a process termed segmentation, before this new generation of invasive merozoites breaks out of the host cell and into the bloodstream through a lytic process referred to as egress. Each of these essential steps in *Plasmodium* asexual blood-stage development is a potential target for therapeutic interventions that would inhibit growth of the parasite and thereby treat or prevent the disease.

Protective immunity to malaria can be acquired through continuous exposure to infection and is associated with the induction of antibodies against a range of parasite proteins (1–3). Antigens capable of eliciting protective immune responses are of interest as prospective components of a greatly needed malaria vaccine. One such candidate is P. falciparum protein PF3D7_1021800, which was named schizont egress antigen-1 (SEA1) after being identified as a potential target of protective antibodies in children exposed to malaria (4, 5) and in vaccine studies in mice (4–6). Despite the potential for SEA1 as a vaccine antigen, a consensus on the function of the protein in the parasite is lacking. Previous studies have generated conflicting conclusions suggesting that SEA1 plays a role either in merozoite egress (4) or in the mitotic division of parasite nuclei as a functional homologue of mammalian centromere protein C (CENP-C) (7, 8). Hence, further investigation of SEA1 function is required to establish whether and how it could be targeted by novel antimalarial interventions.

Both mitosis and subsequent egress are essential processes for the parasite life cycle and the molecular mechanisms underpinning them have been the subject of detailed previous study, but there remain significant gaps in our understanding. Merozoite egress from the infected RBC is a highly regulated process initiated by the activation of the parasite cGMP-dependent protein kinase (PKG), which directly or indirectly mediates the phosphorylation of multiple parasite proteins (9). Pharmacological inhibition or genetic disruption of PKG leads to a complete block in egress (10, 11), suggesting that one or more of the phosphorylation events regulated by PKG are essential for egress. PKG activation leads within minutes to the intracellular discharge of a protease called SUB1, resulting in a series of proteolytic processing events that culminate in rapid dismantling of the parasitophorous vacuole (PV) and host RBC membranes (10, 12). Despite these insights, it remains unknown which of the many PKG-mediated phosphorylation events are required to regulate this cascade or whether additional PKG-dependent processes operate in parallel to control egress. A putative function of SEA1 in egress has been suggested based on the apparent ability of anti-SEA1 antibodies to inhibit schizont rupture (4) as well as evidence that SEA1 is one of at least 69 schizont proteins that are phosphorylated following activation of PKG (9).

While egress is uniquely essential to organisms with intracellular life cycles, mitotic cell division is fundamental to the survival and reproduction of all eukaryotes. Key features of mitosis are highly conserved. Briefly, DNA is replicated and microtubular organizing centers (MTOCs) duplicate at the nuclear periphery. Spindle fibers emanate from these MTOCs and bind to chromosomes via kinetochore proteins that assemble at centromeres. The sister chromatids then segregate and are surrounded by separate nuclear envelopes. P. falciparum asexual replication occurs through a very divergent form of mitotic cell division called schizogony; while eukaryotic cells typically divide via repeated rounds of DNA replication, nuclear division, and cytokinesis, schizogony involves asynchronous DNA replication events, producing a multinucleate cell that is then partitioned into up to 30 mononucleated daughter merozoites, with cytokinesis taking place only at the end of the cycle prior to egress (13–15). The merozoites form around a central food vacuole from which they detach during egress, leaving a structure known as the residual body (15). Mitosis in schizogony is additionally atypical in that there is an absence of chromosome condensation, and the nuclear envelope appears to remain intact while sister chromatids separate (14). These striking features make schizogony both an intriguing process and one that could be targeted by novel therapeutics with minimal risk of toxicity to the host. SEA1 is implicated in schizogony on the basis of bioinformatic and biochemical evidence that it is the P. falciparum homologue of mammalian CENP-C, an essential component of the complex that recruits kinetochore proteins at mitosis (7, 8, 16). SEA1 was shown to associate with P. falciparum centromeres and could genetically complement Saccharomyces cerevisiae lines possessing loss-of-function mutations in the yeast homologue of CENP-C (7). Given the implied localization of SEA1 to the parasite nucleus, these findings raise questions about the proposed function of SEA1 in egress and the potential for SEA1-specific antibodies to interfere with that function.

In light of the conflicting published evidence, here we have used epitope tagging and a robust conditional gene disruption system to further investigate the function(s) of SEA1 in asexual blood stages of P. falciparum. Our results firmly support an essential role for SEA1 in parasite nuclear segregation that is difficult to reconcile with a mechanistic role in egress.

## RESULTS

### Conditional disruption of P. falciparum *SEA1* demonstrates its essentiality.

**FIG 1:**
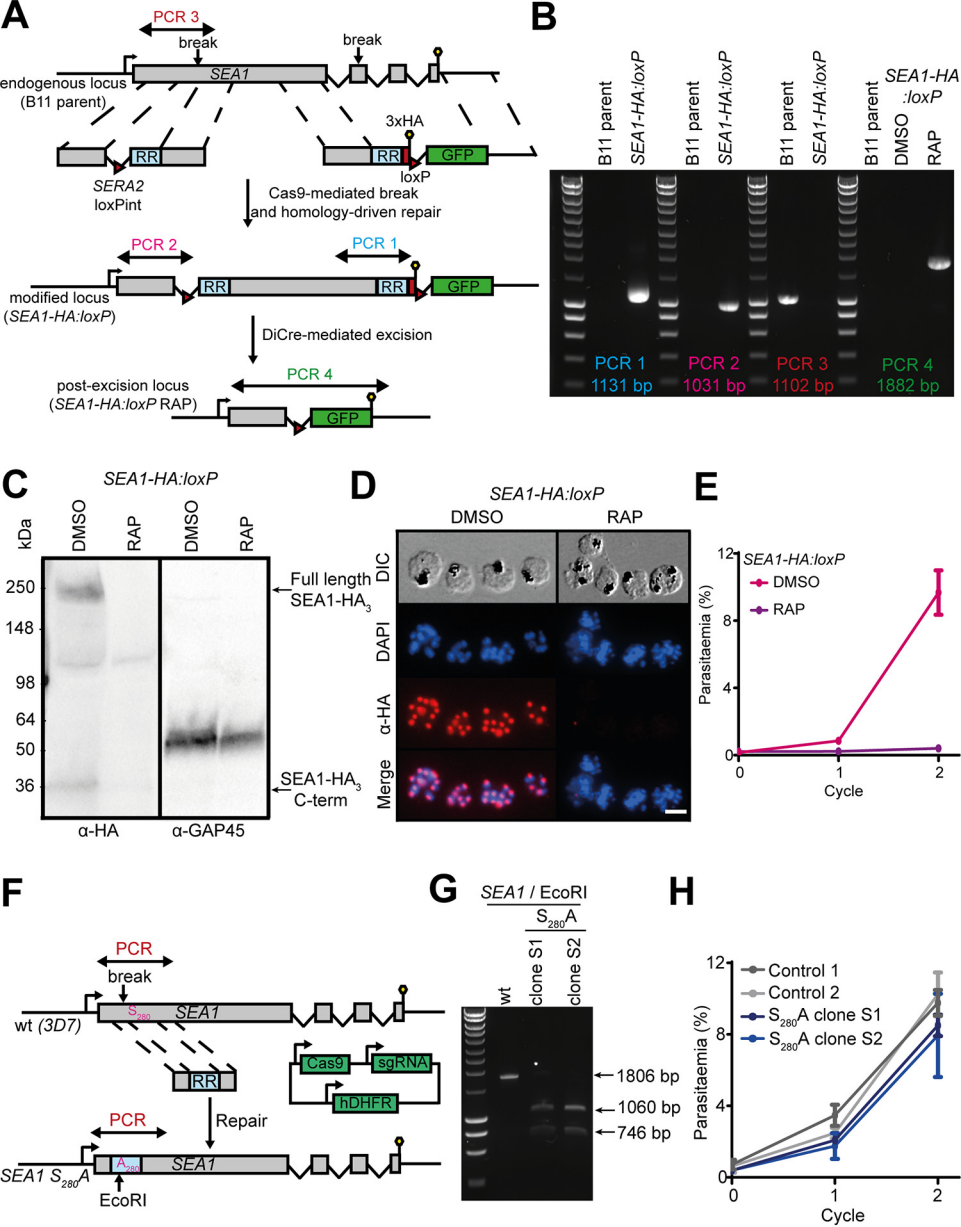
Epitope tagging and conditional disruption of the P. falciparum *SEA1* gene confirms an essential role in asexual blood-stage parasite growth. (A) Schematic showing generation of the *SEA1-HA*:*loxP* line and RAP-induced gene disruption. Double-headed arrows indicate the regions targeted for amplification by diagnostic PCR. Red arrowheads, *loxP* sites. Lollipops, stop codons. RR denotes a recodonized region, and GFP denotes green fluorescent protein. Correct excision was expected to result in the expression of GFP fused to a severely truncated form of SEA1. However, GFP expression was not detectable by fluorescence microscopy. (B) PCR verifying the expected gene modifications and efficient excision of the floxed segment upon RAP treatment of an *SEA1-HA*:*loxP* clone. Amplified regions are illustrated in panel A. (C) Western blot detection of full-length SEA1-HA_3_ (∼250 kDa) along with a putative N-terminal processed fragment (∼30 kDa) in extracts of *SEA1-HA*:*loxP* schizonts and loss of the signals upon RAP treatment. The right-hand panel shows the same samples probed for GAP45 (PF3D7_1222700) as a loading control. (D) IFA showing epitope tagging and DiCre-mediated disruption of SEA1-HA_3_ in schizonts. Over 99% of all RAP-treated *SEA1-HA*:*loxP* trophozoites examined by IFA were HA negative. Scale bar, 10 μm. (E) Growth curves showing that RAP treatment of *SEA1-HA*:*loxP* parasites severely impaired their replication. (F) Schematic representation of Cas9-mediated generation of *SEA1-HA*:*loxP* S_280_A mutant parasites. Double-headed arrows indicate regions targeted for diagnostic PCR in panel G. RR, recodonized region; lollipops, stop codons. (G) PCR verifying correct integration of the recodonized region, including the S_280_A mutation and EcoRI restriction site. The region targeted for PCR amplification is indicated in panel F. (H) Growth curves comparing proliferation of *SEA1-HA*:*loxP* S_280_A parasites and control parental *3D7* parasites.

SEA1 is encoded by the single-copy 6,744-bp *PF3D7_1021800* gene and is predicted to encode a large (∼244 kDa) protein product lacking transmembrane domains or a secretory signal peptide. We used an established conditional gene disruption approach to address the role of SEA1 in asexual blood-stage malaria parasites. To do this, we used a marker-free Cas9-based strategy in a P. falciparum line that expresses a rapamycin (RAP)-inducible Cre recombinase (DiCre) (17) to add both a C-terminal hemagglutinin 3 (HA_3_) epitope tag and a *loxP* site to the 3′ end of the *PF3D7_1021800* gene. In a second manipulation, we replaced the first intron of *PF3D7_1021800* with a *SERA2loxPint* module (18) (Fig. 1A), generating a modified parasite line here referred to as *SEA1-HA*:*loxP* (i.e., harboring an HA_3_-tagged SEA1 gene in which a large segment of the coding sequence was flanked by *loxP* sites). Integration of the modifying constructs was confirmed by diagnostic PCR (Fig. 1B). Successful epitope tagging of SEA1 was demonstrated by Western blotting (Fig. 1C) and immunofluorescence assay (IFA) (Fig. 1D) with an anti-HA monoclonal antibody. Treatment of the *SEA1-HA*:*loxP* parasites with RAP led to rapid, efficient excision of the floxed sequence (Fig. 1B) and loss of expression of tagged protein in at least 99% of parasites by the end of the erythrocytic cycle of treatment (cycle 0) (Fig. 1C and D). Growth assays showed that the resulting SEA1-null parasites failed to proliferate in culture (Fig. 1E), indicating an essential function for SEA1 in asexual blood stages.

### PKG-dependent phosphorylation of SEA1 Ser_280_ is not required for its function.

Activation of the malaria parasite cGMP-dependent protein kinase PKG, a central regulator of parasite egress (10), causes the phosphorylation of a range of target proteins in asexual blood-stage P. falciparum schizonts (9). SEA1 is one of the reported target proteins, with a single phosphorylation site at Ser_280_. These previous data linking SEA1 to egress prompted us to investigate whether PKG-dependent phosphorylation of SEA1 S_280_ contributes to regulation of egress (9). To test this, we used Cas9-enhanced homologous recombination to directly generate mutant parasites in which the *PF3D7_1021800* Ser_280_ codon was replaced by an Ala codon (SEA1 S_280_A) (Fig. 1F). The mutant SEA1 S_280_A line was readily generated, and growth assays showed that the parasites replicated at wild-type rates *in vitro* (Fig. 1G and H). This led us to conclude that PKG-dependent phosphorylation of SEA1 Ser_280_ does not play an important role in asexual blood-stage replication, SEA1 function, or regulation of egress.

### SEA1 localizes to parasite nuclei and associates with nuclear proteins.

**FIG 2:**
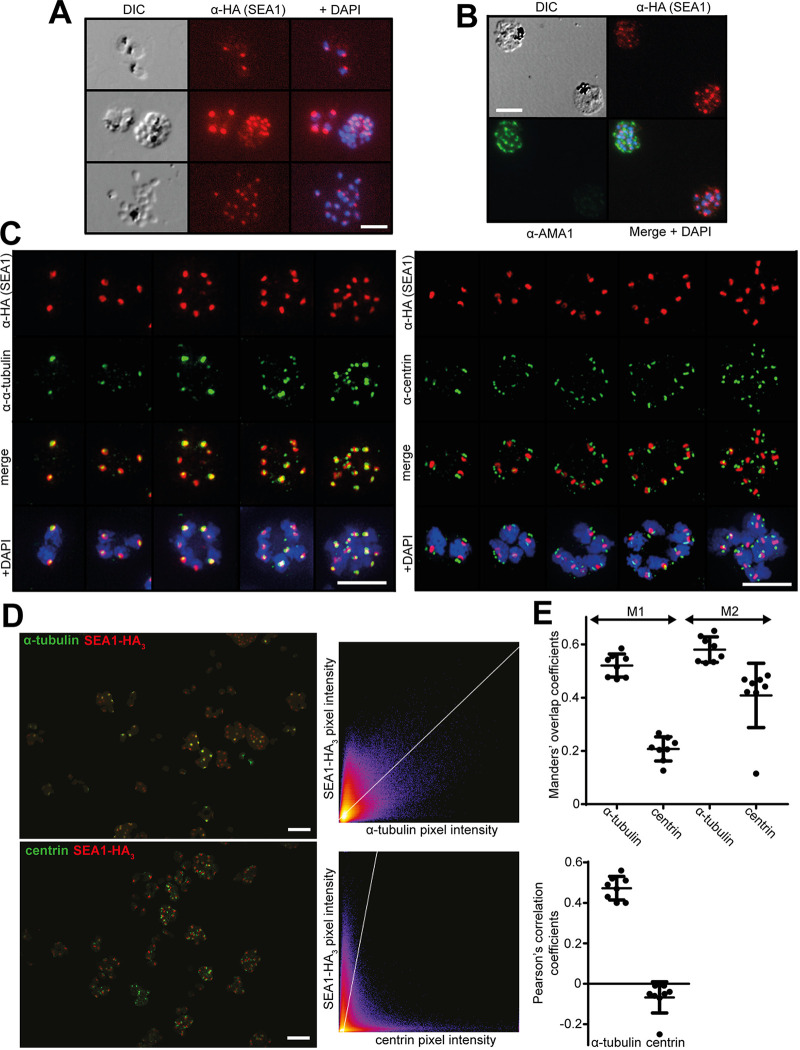
Immunolocalization of SEA1 to a nuclear location. (A) IFA showing localization and relative intensity of SEA1-HA_3_ signals in trophozoite (top)-, schizont (middle)-, and merozoite (bottom)-stage parasites. Scale bar, 10 μm. DIC, differential interference contrast. (B) IFA showing localization of SEA1-HA_3_ and AMA1 in schizonts. The HA signal is strongest in less mature schizonts (bottom right) than in very mature segmented schizonts that express the late-stage marker protein AMA1 (top left). Scale bar, 10 μm. (C) Superresolution immunofluorescence images showing localization of SEA1-HA_3_ in proximity to other proteins at the nuclear periphery. SEA1-HA_3_ loci are closely flanked by sometimes twinned α-tubulin signals (left) and, slightly further away, by often twinned signals for the centrosome marker centrin (right). Scale bars, 5 μm. (D) Example superresolution two-color overlay images of the maximum intensity projections of one pair of the fields of view used in colocalization analyses (left) with their corresponding pixel identity scatterplots (right). Scale bars, 5 μm. (E) Cooccurrence and correlation coefficients from analysis of SEA1-HA_3_, centrin, and α-tubulin signals (including the examples shown in panel D). Manders’ coefficient M1 describes the proportion of green signal (centrin or α-tubulin) that cooccurs with red (SEA1-HA_3_). M2 describes the proportion of red signal that cooccurs with green and shows significantly more cooccurrence of SEA1-HA_3_ with α-tubulin than with centrin (*P = *0.0022, unpaired *t* test). Correlation analysis (below) also shows closer association between SEA1-HA_3_ and α-tubulin signals than between SEA1-HA_3_ and centrin signals. Mean values from eight different fields of view for each marker protein are shown. Error bars, standard deviations (SD).

**FIG 3:**
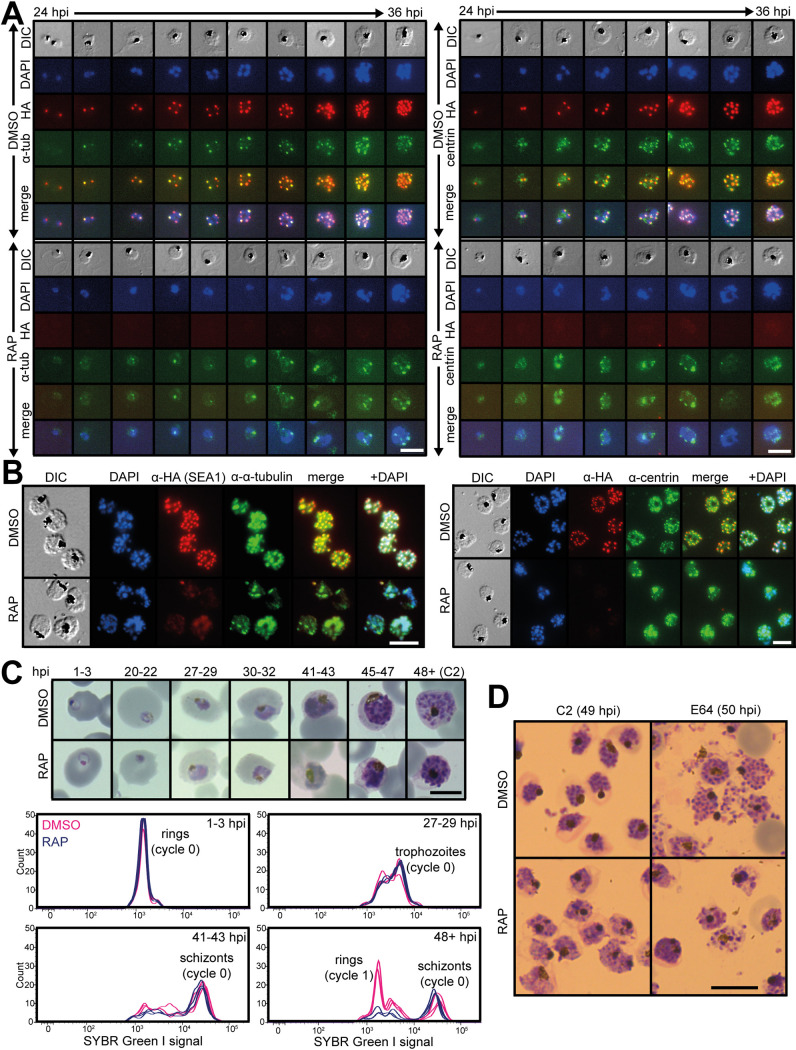
SEA1-null parasites complete DNA replication but display unusual nuclear morphology. (A) IFA showing localization of SEA1-HA_3_ in proximity to α-tubulin and centrin throughout the development of *SEA1-HA*:*loxP* trophozoites/early schizonts. SEA1-HA_3_ is absent in RAP-treated parasites. Scale bar, 10 μm. (B) IFA showing localization of SEA1-HA_3_ in proximity to α-tubulin and centrin in *SEA1-HA*:*loxP* schizonts (∼45 h postinvasion, hpi). Scale bar, 10 μm. (C) Images from Giemsa-stained thin films showing the development of *SEA1-HA*:*loxP* parasites throughout the cycle of treatment with DMSO or RAP (cycle 0). Selected time points (hpi) are accompanied by plots displaying the DNA content of each infected RBC, as determined by SYBR green I staining and flow cytometry. Scale bar, 5 μm. (D) Images from Giemsa-stained thin films showing mature *SEA1-HA*:*loxP* parasites formed at the end of the cycle of treatment with DMSO or RAP (cycle 0). Egress was blocked in these samples by treatment from 45 to 49 hpi with the PKG inhibitor compound 2 (left) and then with the cysteine protease inhibitor E64 (right) from 49 to 50 hpi. Scale bar, 10 μm.

To seek to understand the essential function of SEA1, we used the epitope-tagged protein expressed by the *SEA1-HA*:*loxP* parasites to investigate its subcellular localization. Analysis by IFA showed an SEA1-HA_3_ signal closely associated with the nucleus (Fig. 1D and 2A to C), as previously observed in one study (7) (but in contrast to another [4]). This strong, punctate signal was intense in *SEA1-HA*:*loxP* trophozoites and early schizonts (Fig. 1D and 2A to C) (in which nuclear replication is actively progressing) but was much reduced in very mature segmented schizonts (in which nuclear replication has ceased) that expressed the microneme protein AMA1 (Fig. 2B). Further double-staining analysis using superresolution imaging showed that the SEA1-HA_3_ signal comprised a single focus within each nucleus that was often flanked by twinned signals corresponding to components of the nuclear division apparatus (Fig. 2C to E and 3A and B). The SEA1-HA_3_ foci localized most closely to α-tubulin, which is a component of MTOCs and a marker for mitotic spindles. The signals for centrin, a key component of the centrosome, flanked the SEA1-HA_3_ foci more distally (Fig. 2C to E and 3A and B). We interpreted these results as strongly indicative of a subcellular localization of SEA1 in the dividing nucleus at a position that could be consistent with the previously proposed centromere association (7).

A previous study used an anti-SEA1 antibody to immunoprecipitate α-tubulin from asexual blood-stage parasites (7), indicating that these two proteins associate *in vivo*. We performed similar immunoprecipitation experiments using anti-HA antibodies. Mass spectrometric analysis of the pulldowns detected over 100 proteins associating specifically with the tagged SEA1 (see Table S1 in the supplemental material). While we did not detect α-tubulin in the set of SEA1-HA_3_-associated proteins, this list was particularly enriched with proteins annotated as being localized to the nucleus (Table S1). These data support our localization results, indicating that SEA1 is present in a nuclear or perinuclear location and interacts with other nuclear proteins.

10.1128/mBio.03377-20.4 TABLE S1 Proteins detected by mass spectrometry in anti-HA coimmunoprecipitation analysis of extracts of DMSO- and RAP-treated *SEA1-HA*:*loxP* parasites. Download Table S1, XLSX file, 0.04 MB. Copyright © 2021 Perrin et al. 2021 Perrin et al. https://creativecommons.org/licenses/by/4.0/ This is an open-access article distributed under the terms of the Creative Commons Attribution 4.0 International license.

### Loss of SEA1 results in defective schizogony.

**FIG 4:**
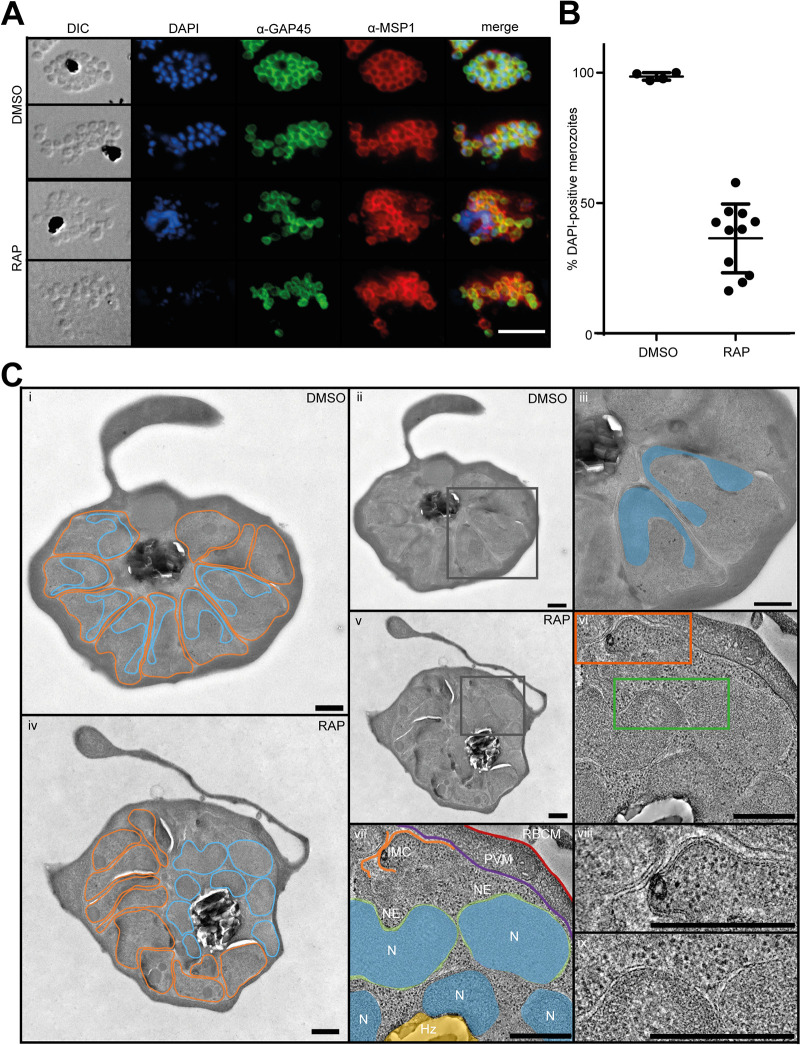
SEA1-null parasites display defective nuclear segregation during schizogony. (A) IFA images showing signals for MSP1 (a marker for the parasite plasma membrane), GAP45 (marking the IMC), and the localization of parasite DNA in mature DMSO- or RAP-treated *SEA1-HA*:*loxP* schizonts and merozoites. Scale bar, 10 μm. (B) Quantification of the proportions of merozoites staining DAPI (DNA)-positive by IFA. At least 50 merozoites were counted per replicate. Totals of 554 and 682 merozoites were analyzed for the DMSO- and RAP-treated populations, respectively. Error bars, SD. (C) Electron micrographs of mature segmented DMSO- and RAP-treated *SEA1-HA*:*loxP* schizonts. (i) Example micrograph of a control (DMSO-treated) *SEA1-HA*:*loxP* schizont with merozoites outlined in orange and nuclei outlined in blue. (ii) Micrograph shown in panel i, highlighting the region shown in more detail in panel iii (black box). (iii) Region of control schizont indicated in panel ii, with C-shaped nuclei in two neighboring merozoites shaded in blue. (iv) Example micrograph of an *SEA1*-null schizont with merozoites and nuclei outlined as in panel i. All merozoites appear nonnucleated in this view. (v) Micrograph shown in panel iv, highlighting the region of tilt-series acquisition shown in panels vi to ix. (vi) Averages from 20 slices from the central portion of a tomogram of the region within *SEA1*-null schizont indicated in panel v (see also Movie S1). IMC and nuclear envelope features are present in orange and green boxes and shown in more detail in panels viii and ix, respectively. (vii) An annotated version of the tomogram highlighting the following cellular features: N (blue), nucleus; NE (green), visible nuclear envelope; Hz (yellow), hemozoin crystals in food vacuole; IMC (orange), visible IMC underlying the cytoplasmic membrane of a partially formed merozoite; PVM (purple), parasitophorous vacuole membrane; and RBCM (red), host RBC membrane. (viii) Region from the tomogram in panel vi showing more detail of the IMC formed in the *SEA1*-null schizont. (ix) Region from the tomogram in panel vi showing more detail of nuclear envelopes surrounding the *SEA1*-null nuclei that have not segregated into merozoites. Scale bars, 500 nm.

To understand the essential function of SEA1, we examined the development of RAP-treated *SEA1-HA*:*loxP* (SEA1-null) parasites. SEA1-null ring-stage parasites were able to progress through the trophozoite stage, replicate their DNA, and form schizonts (Fig. 3A to D). Close inspection of mature SEA1-null schizonts by IFA showed the presence of plasma membranes and underlying inner membrane complexes (IMCs), indicated by MSP1 and GAP45 staining, respectively, delineating each merozoite (Fig. 4A and Fig. S1A). However, the distribution of the DNA in the mutant parasites was markedly different from that in wild-type schizonts, with Giemsa- or 4′,6-diamidino-2-phenylindole (DAPI)-stained nuclear material accumulating in large diffuse aggregates rather than the defined, punctate signal normally observed in mature wild-type merozoites (Fig. 3D and 4A and B and Fig. S1A). Additionally, a large proportion of the DNA appeared to be present within an expanded food vacuole rather than within daughter merozoites in SEA1-null schizonts (Fig. 4A and B and Fig. S1A).

10.1128/mBio.03377-20.1 FIG S1 Additional images of mature segmented *SEA1-HA*:*loxP* schizonts, showing mislocalization of DNA and nuclei in SEA1-null schizonts. (A) Additional IFA images showing the localization of MSP1, GAP45, and parasite DNA in mature DMSO- or RAP-treated *SEA1-HA*:*loxP* schizonts. Scale bar, 10 μm. (B) Overviews of two additional electron micrographs of SEA1-null schizonts (left) with the indicated regions shown in more detail in right-hand panels i and ii. In these panels, discernible merozoites are outlined in orange. Nuclei or clusters of nuclei are outlined in blue. Scale bars, 500 nm. Download FIG S1, TIF file, 1.0 MB. Copyright © 2021 Perrin et al. 2021 Perrin et al. https://creativecommons.org/licenses/by/4.0/ This is an open-access article distributed under the terms of the Creative Commons Attribution 4.0 International license.

10.1128/mBio.03377-20.6 MOVIE S1 Electron tomography of the region of the RAP-treated *SEA1-HA*:*loxP* schizont shown in Fig. 3C. Dual-axis tilt series were acquired from −60° to +60° with an increment of 2°. Download Movie S1, MPG file, 5.8 MB. Copyright © 2021 Perrin et al. 2021 Perrin et al. https://creativecommons.org/licenses/by/4.0/ This is an open-access article distributed under the terms of the Creative Commons Attribution 4.0 International license.

To investigate these cellular features in more detail, we carried out electron tomography on high-pressure-frozen, freeze-substituted plastic sections of mature, segmented *SEA1-HA*:*loxP* schizonts (Fig. 4C and Fig. S1B and Movie S1). Control schizonts typically comprised a set of fully-formed merozoites, each with a C-shaped nucleus, positioned proximal to the site of attachment to the central food vacuole (Fig. 4Ci to iii). This was in stark contrast to mature SEA1-null schizonts, where merozoites were equipped with all the organelles typical of mature schizonts, but, strikingly, most were nonnucleated (Fig. 4Civ and v and Fig. S1). The misplaced nuclei, bounded by intact nuclear envelopes and coated with ribosomes, were observed clustered together with the hemozoin crystal within or adjacent to the food vacuole in a ribosome-filled cytoplasm (Fig. 4Civ to ix). In this region, we also observed IMC structures outside the defined merozoites (Fig. 4Cvi to viii), indicating that the absence of SEA1 leads to delayed, impaired, or aborted segmentation. Together with the IFA evidence, these observations showed a severe defect in the localization and segregation of nuclei in the SEA1-null mutants.

### SEA1-null merozoites egress aberrantly and are not viable.

**FIG 5:**
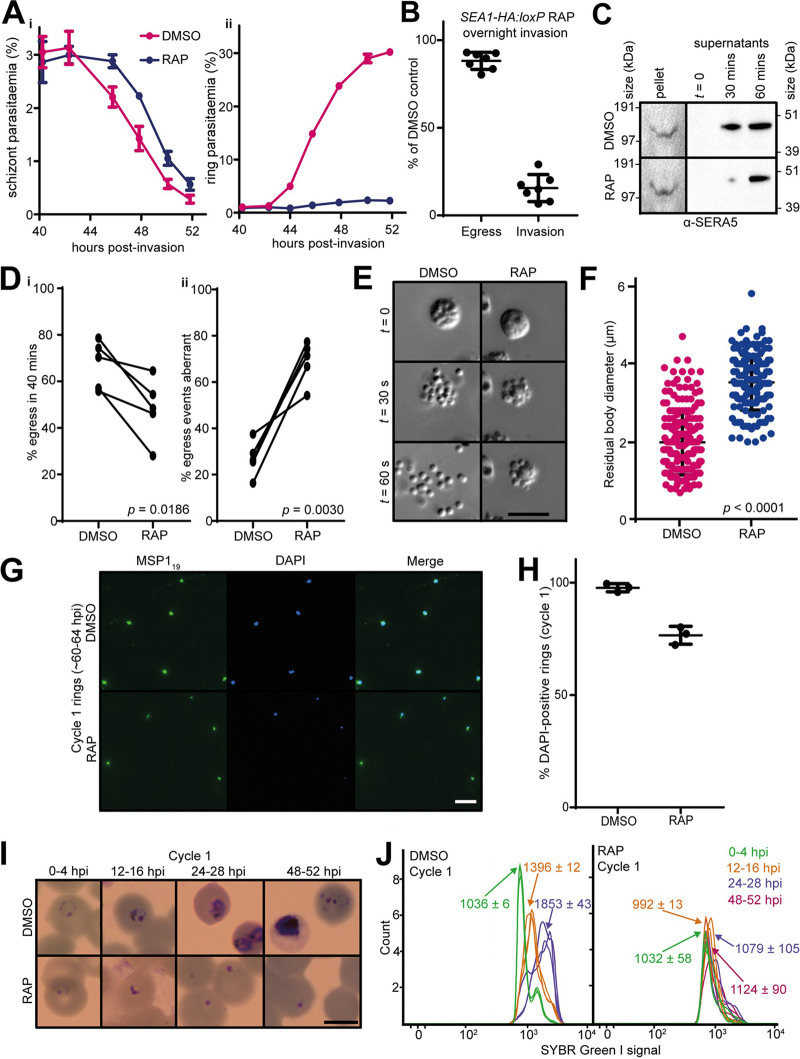
SEA1-null parasites egress aberrantly and fail to proliferate. (A) Time course showing the results of monitoring schizont (i) and ring (ii) parasitemia around the expected time of egress of DMSO- and RAP-treated *SEA1-HA*:*loxP* schizonts. Parasitemia values were measured by flow cytometry. Data points are means from three technical replicates and are representative of four independent experiments. Error bars, SD. (B) Quantification of egress and invasion of RAP-treated *SEA1-HA*:*loxP* parasites relative to controls. Schizonts were enriched, added to fresh RBCs, and left to invade under shaking conditions for at least 8 h before egress and invasion rates were measured by flow cytometry. (C) Western blots monitoring release in culture supernatants of the processed PV protein SERA5 (p50) as a proxy for egress of DMSO- and RAP-treated *SEA1-HA*:*loxP* schizonts. (D, i) Quantification of egress events observed by microscopic monitoring of preparations of highly synchronous DMSO- and RAP-treated *SEA1-HA*:*loxP* schizonts. (ii) Proportion of these egress events in panel i that were aberrant. An egress event was defined as aberrant if more than 3 daughter merozoites remained associated with the residual body (29) and/or if fewer than 3 merozoites were released upon schizont rupture. At least 40 schizonts per condition were observed in each of five matched pairs of videos. *P* values derive from paired *t* tests. (E) Stills selected from time-lapse video microscopy (see Movie S1) showing aberrant egress and an enlarged residual body in RAP-treated *SEA1-HA*:*loxP* parasites compared to DMSO-treated controls. Scale bar, 10 μm. (F) Quantification of residual body size measured following egress of DMSO- or RAP-treated *SEA1-HA*:*loxP* schizonts. More than 150 residual bodies from a total of five videos per condition were measured, and each point represents an individual residual body. Mean values and standard errors of the means are indicated, and *P* values were derived from Student's *t* test. (G) IFA showing control and SEA1-null ring-stage parasites from the cycle following DMSO/RAP treatment (cycle 1). Ring-stage parasites were identified by staining with an antibody against the C-terminal fragment of MSP1 (MSP1_19_). Scale bar, 10 μm. (H) Quantification of the proportions of MSP1_19_-positive cycle 1 rings possessing detectable DNA by IFA (DAPI positive). Totals of 549 and 301 merozoites were analyzed for the DMSO- and RAP-treated populations, respectively. Error bars, SD. (I) Images from Giemsa-stained thin films showing the fate of *SEA1-HA*:*loxP* parasites that successfully invade following the cycle of treatment with DMSO or RAP. Rings derived from the RAP-treated (SEA1-null) merozoites failed to develop. Scale bar, 5 μm. (J) Flow cytometry plots showing SYBR green I fluorescence (indicating DNA content) associated with parasite-infected RBC over the course of the erythrocytic cycle following treatment of *SEA1-HA*:*loxP* parasites with DMSO or RAP (cycle 1). Labels indicate the mean fluorescence intensity of each population ± SD from three replicates. Parasites derived from the RAP-treated (SEA1-null) population failed to develop in cycle 1, consistent with the microscopic images shown in panel H.

To assess the effects of the SEA1-null defect on merozoite egress, we monitored the fate of highly synchronized schizont populations using established approaches to measure schizont rupture and RBC invasion by the released merozoites. Despite the morphological defects in SEA1-deficient schizonts, they did undergo rupture, with overall proportions of SEA1-null schizonts undergoing egress at around 90% of those observed in controls (Fig. 5Ai and 5B). We observed proteolytically processed SERA5 in cell culture supernatants (Fig. 5C), indicating that SUB1 discharge and activity occur and that the PV and RBC membranes break in both DMSO- and RAP-treated *SEA1-HA*:*loxP* schizonts. Schizont rupture in the RAP-treated *SEA1-HA*:*loxP* parasites was slightly delayed compared to that of controls (Fig. 5Ai, C, and D), consistent with observations reported for SEA1-knockdown parasites (4). However, close inspection by time-lapse video microscopy showed that the SEA1-null schizonts ruptured atypically, often failing to release merozoites effectively (Fig. 5D and 5E and Movies S2, S3, and S4). The residual bodies that remained following egress of the SEA1-null parasites were significantly larger (on average, ∼1.8-fold greater in diameter) than those from control parasites (Fig. 5F) and contained DNA (Movies S2 and S3), consistent with the observations of DNA and nuclear mislocalization we made by light microscopy, IFA, and electron tomography (Fig. 3D and 4A to C). Invasion was severely impaired in the absence of *SEA1* (Fig. 5Aii and B), and most of the SEA1-null ring-stage parasites that formed subsequently contained some detectable DNA (Fig. 5G and H). However, these rings were unable to develop further (Fig. 5I and J). These observations indicate that correct partitioning of DNA into individual merozoites is critical to their subsequent development. We concluded that impaired development of SEA1-null parasites leads indirectly to atypical egress and the release of defective merozoites that cannot complete another cycle of replication.

10.1128/mBio.03377-20.7 MOVIE S2 Time-lapse video microscopy of DMSO- and RAP-treated *SEA1-HA*:*loxP* schizonts undergoing egress. Deficiencies in merozoite release and unusually large residual body dimensions are evident in RAP-treated (SEA1-null) parasites. Download Movie S2, MPG file, 6.2 MB. Copyright © 2021 Perrin et al. 2021 Perrin et al. https://creativecommons.org/licenses/by/4.0/ This is an open-access article distributed under the terms of the Creative Commons Attribution 4.0 International license.

10.1128/mBio.03377-20.8 MOVIE S3 Time-lapse video microscopy of a Hoechst-stained DMSO-treated *SEA1-HA*:*loxP* schizont undergoing egress. Download Movie S3, MPG file, 1.7 MB. Copyright © 2021 Perrin et al. 2021 Perrin et al. https://creativecommons.org/licenses/by/4.0/ This is an open-access article distributed under the terms of the Creative Commons Attribution 4.0 International license.

10.1128/mBio.03377-20.9 MOVIE S4 Time-lapse video microscopy of a Hoechst-stained RAP-treated (SEA1-null) *SEA1-HA*:*loxP* schizont undergoing egress, producing an enlarged residual body that contains DNA. Download Movie S4, MPG file, 1.9 MB. Copyright © 2021 Perrin et al. 2021 Perrin et al. https://creativecommons.org/licenses/by/4.0/ This is an open-access article distributed under the terms of the Creative Commons Attribution 4.0 International license.

## DISCUSSION

Our results demonstrate that SEA1 is an essential P. falciparum asexual blood-stage protein that plays an important role in orchestrating the correct partitioning of DNA into merozoites. Our finding that *SEA1* is an essential P. falciparum gene is consistent with previous results of others showing that knockdown of *SEA1* expression severely impairs parasite replication (4). A recent high-throughput transposon-based insertional mutagenesis screen for essential P. falciparum genes suggested that it was possible to mutate the *SEA1* gene without impairment of parasite growth (19). In this particular case, however, the insertion observed was close to the 3′ end of the gene (its position corresponding to residues 1766 to 2248), such that at least 78% of the gene could still be translated. Taken together, these results indicate that while SEA1 is an essential protein, residues close to the C terminus are not critical for its function.

Using direct mutagenesis of *SEA1*, we have demonstrated that the reported PKG-dependent phosphorylation of SEA1 at Ser_280_ is not essential for its function in asexual blood-stage *Plasmodium* parasites. In parallel work, we also found that PKG-dependent phosphorylation sites (9) in eight additional proteins thought to function in merozoite egress and/or invasion are nonessential (see Fig. S2 and S3 in the supplemental material). These proteins include GAP45, a component of the parasite’s essential invasion motor complex (17) (Fig. S3). Together, these results indicate that many of the specific phosphorylation events mediated via PKG activation do not individually contribute significantly to the critical function of PKG. Further investigation of PKG activity will be required to determine which phosphorylation events in which protein substrates coordinate the essential processes of microneme and exoneme secretion (10), Ca^2+^ signaling (20), and egress (10, 11, 21), as well as to determine how PKG activity also regulates RBC invasion (9).

10.1128/mBio.03377-20.2 FIG S2 PKG-dependent phosphosites are not essential for P. falciparum asexual blood-stage growth. (A) Diagnostic PCR verifying the Cas9-mediated replacement of regions within eight P. falciparum genes, so as to introduce the indicated Ser-to-Ala mutations at PKG-dependent phosphorylation sites. Three of these replacement events remove introns from the native gene, resulting in PCR products of reduced length in each of two modified clones compared to the parental 3D7 control line (wt). Five of these events do not change the number of bases present at the locus but do introduce the indicated restriction site within the modified locus. Protein name abbreviations: A, *ARO*, *PF3D7_0414900*; C, Coronin, *PF3D7_1251200*; D, *DynR*, *PF3D7_1434500*; I, *IMC1g*, *PF3D7_0525800*; N, *NAB2*, *PF3D7_0623100*; P, *Pf332*, *PF3D7_1149000*; S, *SEA1*, *PF3D7_1021800;* Y, *YOP1*, *PF3D7_0316700.* (B) Growth curves showing *in vitro* proliferation of the phosphosite mutants shown in panel A alongside wild-type parasite controls. The mean from three replicates is plotted. Error bars, SD. (C) Phosphosite mutant multiplication rates from a single cycle of intraerythrocytic growth, shown as a ratio to that of 3D7 controls. The mean from at least three replicates is plotted. Error bars, SD. Download FIG S2, TIF file, 0.4 MB. Copyright © 2021 Perrin et al. 2021 Perrin et al. https://creativecommons.org/licenses/by/4.0/ This is an open-access article distributed under the terms of the Creative Commons Attribution 4.0 International license.

10.1128/mBio.03377-20.3 FIG S3 Conditional substitution of PKG-dependent phosphosites in GAP45. (A) Schematic representation of the generation and use of the *GAP45lox_S_149/156_A* line, which, upon treatment with RAP, results in the conversion of GAP45 Ser 149 and 156 to Ala and the introduction of an HA_3_ tag. Purple lines represent Ser-to-Ala mutations, lollipops represent stop codons, RR denotes recodonized regions, and red and green arrows indicate the annealing sites of the primers used in panel B. (B) PCR showing successful integration of the *GAP45lox_S_149/156_A* transgene in one clone and successful excision of sequence between *lox* sites upon treatment with RAP. (C) Western blots showing allelic switch of GAP45 to GAP45-HA_3_ upon treatment with RAP. The left-hand panel shows the signals detected using an anti-HA monoclonal antibody, and the right-hand panel shows the same samples stained using an anti-GAP45 antibody. (D) IFA showing localization of GAP45 and GAP45-HA_3_ in control and RAP-treated *GAP45lox_S_149/156_A* schizonts. Over 99% of all RAP-treated *GAP45lox_S_149/156_A* schizonts examined by IFA were HA positive, whereas all DMSO-treated schizonts were HA negative. Scale bar, 5 μm. (E) Growth curves showing *in vitro* proliferation of the *GAP45lox_S_149/156_A* line. Treatment with RAP and the resultant tagging and introduction of phosphosite mutations do not impair parasite growth. The mean from three replicates is plotted. Error bars, SD. Download FIG S3, TIF file, 1.0 MB. Copyright © 2021 Perrin et al. 2021 Perrin et al. https://creativecommons.org/licenses/by/4.0/ This is an open-access article distributed under the terms of the Creative Commons Attribution 4.0 International license.

SEA1 was previously designated an egress antigen based on *in vitro* data, indicating that egress was delayed upon protein knockdown and that anti-SEA1 antibodies appeared to block schizont rupture (4). However, a more recent study did not replicate the growth-inhibitory effects of anti-SEA1 antibodies (22), and our present data demonstrate that, despite severe morphological defects, SEA1-null schizonts do undergo egress. Vaccination studies appear to indicate that an anti-SEA1 immune response can elicit some protection from parasitemia (4, 6), but based on our data, we suggest that this is unlikely to be explained by any egress-blocking capacity of anti-SEA1 antibodies.

SEA1 was previously proposed to be a functional homologue of the mammalian centromere-binding protein CENP-C, a component of the kinetochore complex that links centromeres to microtubules as chromosomes are segregated during mitosis (7). CENP-C knockout phenotypes in vertebrate cells include delayed mitosis (23) and chromosome mis-segregation (23, 24). Our data showing that (i) SEA1 localizes in close proximity to MTOC components within each parasite nucleus, (ii) that the SEA1 foci often lie between twin puncta obtained with antibodies expected to highlight microtubule spindles (α-tubulin) and centrosomes (centrin), (iii) that SEA1 expression appears to be highest in nuclei during the actively dividing trophozoite and early schizont stages, and (iv) that SEA1-null parasites fail to properly segregate replicated DNA into merozoites are collectively consistent with SEA1 playing a role similar to that of CENP-C during schizogony. The small proportion of merozoites released from *SEA1*-knockout schizonts that successfully invade a new RBC subsequently fail to progress from ring to trophozoite stage; we estimate that around 20% of these invasive merozoites did not contain nuclei, and it is quite possible that those that were nucleated did not contain the normal complement of chromosomes, in line with what has been observed in CENP-C-deficient cells in other systems (23, 24). SEA1 has a relatively low level of sequence homology with known CENP-C proteins, but together with the previous data indicating that regions of the P. falciparum SEA1 protein can complement CENP-C deficiencies in yeast (7), our data support the hypothesis that SEA1 plays an essential role at or near P. falciparum kinetochores during schizogony.

The divergent mechanisms by which *Plasmodium* parasites control daughter cell formation and how this is coordinated with DNA replication and nuclear division remain largely unclear. These processes have been much more intensively investigated in Toxoplasma gondii tachyzoites, which reproduce by endodyogeny rather than by schizogony (25). In these parasites, the initiation of daughter cell formation relies on structures emanating from centrosomes, thereby linking nuclear replication to segmentation (26). Similar mechanisms operating in P. falciparum schizonts could explain how the loss of a potential centromere-associated protein could have a downstream effect on the formation of merozoites, although there is a growing body of evidence suggesting that cytoplasmic division in P. falciparum schizonts does not depend on the completion of nuclear division. In particular, recent detailed electron microscopy studies describe the initiation of membrane invagination while nuclei are uncompacted and not fully divided (15), and a very recent study has shown that there may be no mechanism that prevents cytokinesis from taking place in the absence of normal nuclear division (27).

The aberrant morphology of segmented SEA1-null schizonts, typified by incompletely formed merozoites that fail to effectively separate from an expanded residual body, is strikingly similar to that observed by others upon knockdown of a small number of other parasite proteins, including cyclin homologue Cyc1 (PF3D7_1463700) (28), a basal complex component named CINCH (P. falciparum coordinator of nascent cell detachment, PF3D7_0407800) (29), and merozoite organizing protein (MOP, PF3D7_0917000), which has been proposed to be important in defining the apical end of developing merozoites (30). We used affinity purification-mass spectrometry to attempt to obtain insights into SEA1-associated proteins, and the results strongly suggested an association with nuclear proteins. However, it is worth noting that generation of parasite extracts led to degradation of the full-length SEA1-HA_3_ protein to an ∼30-kDa HA-tagged C-terminal fragment (Fig. 1C), which was the predominant species captured by the anti-HA antibodies and identified by mass spectrometry. We suspect that as a result, our experiments did not capture the full profile of proteins potentially interacting with SEA1 *in vivo*. Nonetheless, our data indicate that SEA1 physically associates with CINCH in schizonts, suggesting a functional link between these two proteins. However, differences in the established subcellular localizations of these two proteins (29) and the lack of detection of SEA1 in a published CINCH affinity purification-mass spectrometry study (29) means these data should be interpreted with caution. Similar to our SEA1-null schizonts, MOP and CINCH knockdown mutants can undergo egress despite severe morphological defects (29, 30). Taken together, these phenotypes show that proper merozoite segmentation is not a prerequisite for egress and suggest that the developmental cues that govern merozoite formation and egress are quite distinct.

In conclusion, this work has sought to address inconsistencies arising from previous publications regarding the suggested localization and function of SEA1 and, as such, has established that (i) *SEA1* is an essential parasite gene, (ii) SEA1 localizes to the nucleus of trophozoites and immature schizonts in a manner consistent with it being a centromere-associated protein, (iii) SEA1 function is critically important for nuclear segregation and schizont development prior to egress, and (iv) neither SEA1 nor the completion of segregation are required for merozoite egress, making it unlikely that anti-SEA1 antibody responses could block egress as part of a protective immune response.

## MATERIALS AND METHODS

### P. falciparum culture and transfection.

Transgenic P. falciparum erythrocytic stages were cultured and synchronized in human erythrocytes, as described previously (31). Schizonts were enriched for all transfections, which were performed using an AMAXA 4D Nucleofector and P3 reagent (Lonza). Transfected parasites were selected with WR99210 as described previously (17).

### Generation and conditional disruption of *SEA1-HA*:*loxP*.

The *SEA1-HA*:*loxP* parasite line was generated by Cas9-mediated genome editing of P. falciparum B11 parasites that constitutively express the components of the DiCre system (17). A synthetic *loxP*-containing intron was added close to the 5′ end of the gene using a commercially synthesized repair template (GeneArt; Thermo) comprising a 5′ homology region (bp 201 to 950), a *SERA2loxPint* module (18), approximately 400 bp of recodonized sequence (Table 1), and a 3′ homology region (bp 2642 to 3391). The linearized repair template was cotransfected with a pDC2-based plasmid encoding Cas9 and a single guide RNA (sgRNA) targeted to ATTGTTGAAGAAGAACAATG. The 3′ end of the gene was modified using the same methodology with an sgRNA targeted to GTTGATCCTATAGATGATGG and another synthetic construct comprising a 5′ homology arm (bp 5143 to 5892), a 3′ 30-bp recodonized region corresponding to the final 110 amino acids of coding sequence, an HA_3_ tag sequence followed by a stop codon and *loxP* site, an enhanced GFP reporter gene, and a 994-bp homology region corresponding to the intergenic region downstream of *SEA1*. Clones were obtained by limiting dilution, and successful double modification was confirmed by PCR (Table 2 and Fig. 1A and B) and capillary sequencing.

**TABLE 1:** Synthetic sequences used in modification of the SEA1 gene

SEA1 region	Synthetic sequence^*a*^
*SEA1-HA*:*loxP* 5′ recodonized amino acids L_318_–D_450_	TTACGCGACAAGCGCGGTAAGTATCACAAGCTTGGAGACTACCAGAACATCGAGAACTACCGCAAGACAGGAGAGCACTCGTTCGACTGCATGAACATGAGCGATATTATGCACAGCAACAAGATGAGTCACGTAAACATTATGGACCACATGATCTACAAGGACAACAATAACATGTCTAAGTTAGTGGACACTATCAACTCACGCGAGAAAGACGTCAAGAACTACGATGACAATTTCGAGTCCTACAACAACTTCTTTAAAAACAACAATGACGAGCAGCACATTTGCTTAGAATACGATGACACCTACAATCTTAAGGACACAGTAAAGAACATCATCGTAGAAGAGGAGCAGTGTGGAAAAGGAGTAGCATGCATTTGCGACAAAAATGAGGAC
*SEA1-HA*:*loxP* 3′ recodonized amino acids P_1965_–I_2074_	CCAATCAACAAGTTGGCAGTTTCATCTAACTTGGGTCCACCTAGCAGTATTGGCTCTACTGAGATCCAACCAATCCAGAAAAACTTCAATGACTTTAAGATGAACATCAATGTTTATTGTATCCGTATGGAACCACACGAGAAGTATTCGAGCTACAGCCACAAGAACAACCTTGTGGTTTATATCGACAAAGGTGAGAAGATCAACATCATCATCAATATGAGCAAAACCTACGAGAAGGGCGACTTCTTCTATATTCCCCGCTTCAGCAATTTTCAGATTATTAACGACTCCCGCTGCGACTGCGTCCTGTACGTCTGCCCGTTGATT
SEA1 S_280_A recodonized amino acids E_257_–N_448_ with EcoRI site	GAGAACCAGAAGGATATCATCTATCTGAACAACCTGAACAATATCATGATGGACAAGTACAGCAACT***GCG***CGGACTCGCGCAAGAAAGAGTACTCTCACTTTAACTCCCAA**GAATTC**TCGTACGACAAGTACAGCATGAAGGATAGGATGTTCCTGAAGAACTTATACATGAAGCAGAACCGCCTGCGCGACAAGCGCGGCAAGTACCATAAGTTAGGAGACTACCAGAACATCGAGAATTACCGCAAGACAGGAGAGCACAGCTTCGACTGCATGAACATGTCCGACATCATGCACTCCAACAAGATGAGTCACGTTAACATAATGGACCATATGATCTACAAGGATAACAACAACATGAGTAAGCTAGTAGACACTATTAACTCACGCGAGAAAGACGTAAAGAACTATGATGACAATTTCGAGAGTTACAACAACTTCTTTAAAAACAATAACGACGAGCAGCATATTTGCTTAGAGTATGATGACACTTACAACTTAAAGGATACTGTAAAGAACATTATCGTGGAGGAGGAGCAGTGCGGTAAAGGAGTTGCTTGCATCTGCGACAAGAAT

a The SEA1 S280A codon substitution is shown in boldface italic, and the resulting new EcoRI site is in boldface.

**TABLE 2:** PCR primers used in the validation of the integration and excision of gene sequences at the SEA1 locus

PCR	Primer sequence^*a*^
*SEA1-HA*:*loxP*-3′’ integration (Fig. 1, PCR1)	F, GGACAAACATGAAATGGATTTGAAC
	R, GCCCATGGCATAGTCCGGGACGTC
*SEA1-HA*:*loxP* 5′ integration (Fig. 1, PCR2)	F, GATGGAAAATAAATACCCAAATGA
	R, CATATATAATAACTTCGTATAATGTATGC
*SEA1* wt specific (Fig. 1, PCR3)	F, GATGGAAAATAAATACCCAAATGA
	R, CATATGATCCATGATATTAACATGGCTC
*SEA1-HA*:*loxP* excision (Fig. 1, PCR4)	F, GATGGAAAATAAATACCCAAATGA
	R, CAATTTATACAAAAATTGTCCTATTTTC
*SEA1* bp 135–1940 (Fig. 2)	F, GAATGAAAACGATGGTATATGTGAA
	R, TCACGTAGCTCATTACTAAGATCCA

a F, forward; R, reverse.

To induce DiCre activity and excise the majority of the *SEA1* gene, ring-stage *SEA1-HA*:*loxP* parasites were treated with 10 nM RAP (Sigma) for 8 to 12 h. Control parasites were treated with vehicle only (1%, vol/vol, dimethyl sulfoxide [DMSO]).

### Generation of SEA1 S_280_A mutants.

SEA1 S_280_A mutants were generated by Cas9-mediated genome editing of P. falciparum 3D7 parasites, using methods similar to those described above. pDC2-based plasmids carrying sgRNA target ATTGTTGAAGAAGAACAATG (clone 1) or ATAGATTAAGAGATAAAAGG (clone 2) were cotransfected with a linear repair construct comprising a 5′ homology arm (bp 286 to 768) followed by a recodonized region (Table 1) and a 3′ homology arm (bp 1345 to 1844). Clones obtained by limiting dilution were validated via the detection of the novel EcoRI site present in the recodonized region (Tables 1 and 2 and Fig. 2) and by capillary sequencing.

### Flow cytometry.

P. falciparum-infected RBC samples were fixed with 0.2% glutaraldehyde in phosphate-buffered saline (PBS) and then stained with SYBR green prior to analysis using a BD Fortessa instrument.

### IFA.

Thin blood films were fixed with 4% paraformaldehyde (PFA) for 30 min and then permeabilized with PBS containing 0.1% Triton X-100 for 10 min. Slides were then blocked for 1 h in PBS containing 4% bovine serum albumin (BSA) before staining with the relevant antibodies and conjugates. These were 3F10 rat anti-HA monoclonal antibody (MAb) (diluted 1:500; Roche), goat anti-rat biotin conjugate (1:1,000), Alexa 594-streptavidin conjugate (1:1,000; Invitrogen), rabbit anti-AMA1 polyclonal antibody (32) (1:500), rabbit anti-GAP45 polyclonal antibody (1:1,000), goat anti-rabbit Alexa 488 conjugate (1:1,000; Invitrogen), mouse anti-α tubulin MAb (1:500; Sigma), 20H5 mouse anti-centrin MAb (1:500; Millipore), 5.2 mouse anti-MSP1_19_ monoclonal antibody (1:1,000), highly adsorbed goat anti-mouse Alexa 488 conjugate (1:1,000; Invitrogen), X509 human anti-MSP1 antibody (1:1,000), and goat anti-human Alexa 594 conjugate (1:1,000; Invitrogen). Slides were mounted with ProLong gold antifade mountant containing DAPI (Thermo). Imaging was performed using a Nikon Eclipse Ni fluorescence microscope fitted with a Hamamatsu C11440 camera.

### Superresolution imaging and colocalization analysis.

To analyze the colocalization of SEA1-HA_3_ with α-tubulin and centrin, Z-stacks (125-nm Z-step) were acquired on an VT-iSIM superresolution imaging system (Visitech International), using an Olympus IX83 microscope, 150×/1.45 Apochromat objective (UAPON150XOTIRF), ASI motorized stage with piezo Z, and Prime BSI Express scientific complementary metal oxide semiconductor camera (Teledyne Photometrics). The microscope was controlled with Micro-Manager v2.0 gamma software (33). Huygens Deconvolution Software (SVI) was used to enhance the signals shown in Fig. 2C. Colocalization analysis was performed on the original images (without deconvolution) using the Coloc 2 plugin in Fiji. A mask image was generated by autothresholding of the DAPI channel (using Otsu’s autothresholding method). After background subtraction, colocalization of SEA1-HA_3_ with α-tubulin and centrin was assessed using Costes’ autothresholding method to calculate Manders’ overlap coefficients M1 and M2 and Pearson’s correlation coefficient (PCC).

### Time-lapse video microscopy.

Egress videos were carried out as described previously (17). Videos were analyzed using Image J.

### SEA1-HA_3_ immunoprecipitation and proteomic analysis by mass spectrometry.

Synchronous *SEA1-HA*:*loxP* parasite lines were treated at ring stage with DMSO or 10 nM RAP and allowed to develop to early schizont stage (∼40 h). Parasites were extracted from RBCs with 0.15% saponin and washed several times with PBS. Ten matched sample pairs were used to produce two pooled samples of ∼200 μl schizonts (DMSO and RAP treated), from which lysates were prepared in radioimmunoprecipitation assay (RIPA) buffer (Thermo). Triplicate samples of lysates were loaded onto anti-HA-coated magnetic beads (Pierce) by incubation at 4°C with rotation for 1 h. Following wash steps, proteins bound to the beads were eluted by incubation in SDS sample buffer containing 100 mM dithiothreitol (DTT) at 95°C for 10 min.

In-gel tryptic digestion was used to produce peptides for analysis by mass spectrometry. These peptides were dried by vacuum centrifugation and resuspended in 40 μl 0.1% formic acid before being loaded onto prepared Evosep tips and injected on a 15-cm column for 44 min using the HCD IT UM method on an Orbitrap Lumos Fusion instrument. Raw files were analyzed using MaxQuant (34) v1.6.12.0 using the iBAQ algorithm and integrated Andromeda peptide search engine (35). Variable modifications of methionine residues (oxidation) and the protein N terminus (acetylation) were permitted, along with fixed modification of cysteine residues (carbamidomethylation). The estimated false discovery rate was set to 1%. The PlasmoDB v28 P. falciparum and Swiss-Prot H. sapiens protein databases were searched to identify peptides. Further data analyses were performed in Perseus v1.4.0.2 and Microsoft Excel.

### Electron microscopy.

Mature *SEA1*-knockout and control schizonts were treated with the PKG inhibitor 4-[7-[(dimethylamino)methyl]-2-(4-fluorphenyl)imidazo[1,2-α]pyridine-3-yl]pyrimidin-2-amine (compound 2) for 4 h to synchronize the parasites at a highly mature developmental stage, Percoll purified, washed with PBS, and fixed for 5 min at 37°C with 1% glutaraldehyde and 3% formaldehyde. Schizonts were washed again, pelleted, and mixed with 20% (wt/vol) dextran in phenol red-free RPMI containing baker’s yeast before freezing with a Leica HPM100 high-pressure freezer. Vitrified cells were freeze-substituted using a Leica electron micrograph (EM) AFS2 into Lowicryl HM20 resin (EMS) with 0.2% (wt/vol) uranyl acetate. One hundred twenty-nanometer sections were cut using a Leica UC7 microtome and mounted onto glow-discharged, carbon-coated, copper London finder grids (EMS). Sections were poststained with 0.2% (wt/vol) uranyl acetate and 4% (wt/vol) lead citrate (EMS). Micrographs and tilt series were acquired using a Model 2040 dual-axis tomography holder (Fischione Instruments) on a Tecnai T12 120-kV transmission electron microscope (FEI) equipped with a 4K-by-4K Ultrascan 4000 charge-coupled device camera (Gatan). Overview images were acquired using a digital micrograph (Gatan). Dual-axis tilt series were acquired from −60° to +60° with an increment of 2° using SerialEM (36) and processed using eTomo (part of IMOD) (37) with fiducial-less alignment by patch tracking.

10.1128/mBio.03377-20.5 TABLE S2 Oligonucleotide sequences used in the generation of phosphosite mutant parasite lines. Amino acid changes (purple) are indicated in boldface italics, and novel restriction sites (yellow) and HA3 epitope tag (green) are in boldface. Download Table S2, DOCX file, 0.02 MB. Copyright © 2021 Perrin et al. 2021 Perrin et al. https://creativecommons.org/licenses/by/4.0/ This is an open-access article distributed under the terms of the Creative Commons Attribution 4.0 International license.

10.1128/mBio.03377-20.10 TEXT S1 Description of the methods used to generate mutations in PKG-dependent phosphosites in eight P. falciparum genes. Download Text S1, DOCX file, 0.01 MB. Copyright © 2021 Perrin et al. 2021 Perrin et al. https://creativecommons.org/licenses/by/4.0/ This is an open-access article distributed under the terms of the Creative Commons Attribution 4.0 International license.
