# Supplementary figures and images for: Malaria Parasite Schizont Egress Antigen-1 Plays an Essential Role in Nuclear Segregation during Schizogony
Source: mBio. 2021 Mar 9;12(2):e03377-20. doi: 10.1128/mBio.03377-20 (PMC8092294; doi:10.1128/mBio.03377-20)

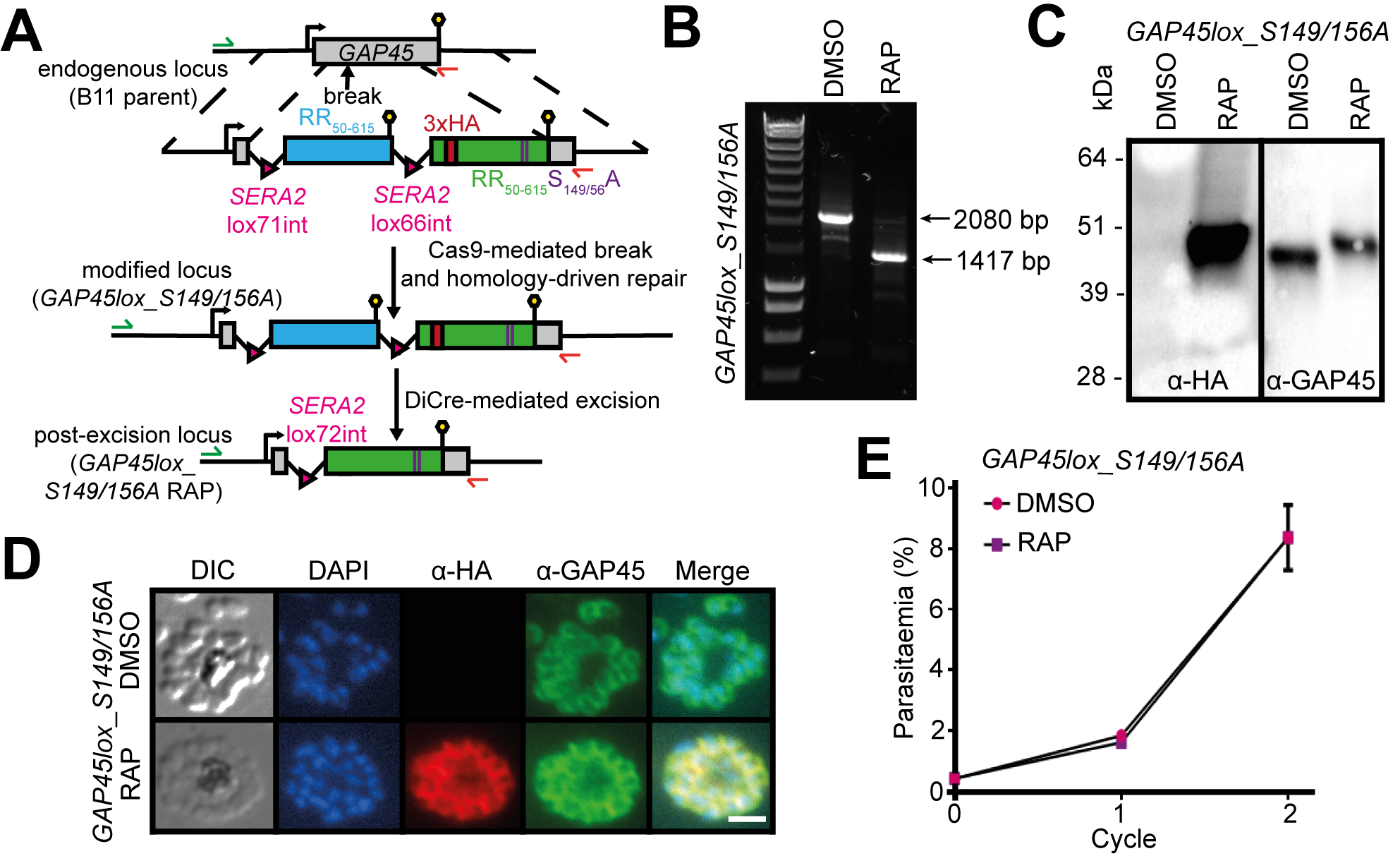

Supplement: FIG S3 [file mBio.03377-20-sf003.tif]
