# Supplementary material for: Malaria Parasite Schizont Egress Antigen-1 Plays an Essential Role in Nuclear Segregation during Schizogony
Source: mBio. 2021 Mar 9;12(2):e03377-20. doi: 10.1128/mBio.03377-20 (PMC8092294; doi:10.1128/mBio.03377-20)
Supplement: TABLE S2 [file mBio.03377-20-st002.docx]

| **Table S2A: sgRNA targeting sequences for the generation of phosphosite mutants** | | |
| --- | --- | --- |
| **Gene** | **gRNA target** | |
| *PF3D7_0414900* (*ARO*) | 1: TATAAAGATGCAATACGTG  2: GTTTTGGGGTCAGCAGCCCA | |
| *PF3D7_1251200* (Coronin) | 1: TGTCATTTGAAAGACAACG  2: GATACAAGGAGAAATAATGG | |
| *PF3D7_1434500* (*DynR*) | 1: CAAAAGGTTAGTAGTGATG  2: GGGTATAGGATTAGGAGAA | |
| *PF3D7_0525800* (*IMC1g*) | 1: GGAAACCCATTATGCAAAGG  2: TTACACAAGAAAACTTCTG | |
| *PF3D7_0623100* (*NAB2*) | 1: TGATACAAGACGACGATCG  2: AGGAGTAGAGGTACGCCAA | |
| *PF3D7_1149000* (*Pf332*) | 1: GAAAAAGTTTCAGTGCATGA  2: CTTGTTGAAGAAGAAGCAT | |
| *PF3D7_1021800* (*SEA1*) | 1: ATTGTTGAAGAAGAACAATG  2: ATAGATTAAGAGATAAAAGG | |
| *PF3D7_0316700* (*YOP1*) | 1: AATGGTACGATAATATATGA  2: GCAATTATTTGTAATGTCGT | |
| *PF3D7_1222700* (*GAP45*) | GAACCTCTTGAACAAGAAC | |
|  | | |
| **Table S2B: Synthetic sequences used in modification of phosphorylation sites** | | |
| **Gene & region** | **Synthetic sequence** | |
| ARO S_33/36_A  Re-codonised amino acids X_11_-H_121_ | TTGCTGTACAAGAACAAGTTGCAGGAATTCGGAATCGAGGGCAGCAAAACCATTCGCAAATTACTG***GCT***TTTACC***GCT***AACGACATTCTTCGCTTCGACAAGGCTTACGATGAGAACGACGTACAGGAATTCGTGAATCTGTGCTCAAGTACTTGTGAGATTGAAAAGCTGGAGGACCGTATGCACCCGTGGGCGGCGGATCCTAAAACAATCGGCGCTCTGAGCGCGACCCAGTTAGCTATTTTGGCCTCTAAAGAGAATGAACCTCACTACAAGGACGCCATCCGCGAAGCCAACGGGATCGCGGTATTCATCAACCTGCTGAAGAGTCAT | |
| Coronin S_570/571_A  Re-codonised amino acids K_436_-K_579_ with HpaI site | AAGAAGAAGGAGACCACTGAAATCCAGGGCGAGATCATGGGTGAGACTAAGTCGTCAATTGAGGCCGACTTCGAGCCGCAGGAGTGCAAGGAGAACAAGAAGGGTAACAAGTTGAACGAGGCCCCTAAGTTCTTGTTCGCATGTGAAGATGTGGAGATCTGCCACCTTAAGGATAATGTTGATGACGACGATTACTTGATC**GTTAAC**GGGACTAACGAACCTTACGAGGAGACTGTTATTAAAACAAACGAGAACGAGAACTACAAAGAGAACAACGACTCAAGCATTCAGTCGATCCGTTCGAACTCCAAGTCCATCGAAAAAAACGACGACGACAACAACAATAACAACAACGATAATACACTTCAGAGCGAGGAGAACGAGGAGCACTTAAAACATATT***GCCGCC***ATCCACGAGGAGAACAACTTTAAA | |
| DynR S_7014_A  Re-codonised amino acids N_6866_-X_7031_ with HindIII site | AATGAACAGAAGAAGAAGGACGAGGACTTCCACAAGGTCGACTTCTTGAAAGGAATCGGTCTGGGTGAAGGAAAGGGAATTAAGAACATCGTGGACGAGGTCGACAACGAGGACATCGACAACATCTACGACGAAAATGAGCAGAAAGTCTCATCTGACGAAGAGCAGGACTTCAATGAGGAGGCAATCGAGGCGAGCTACAACTTCGAGAAGTTTTGCGAGAAGGAGTTCAAGGACATCAACAACTTCGACAACGAAAAG**AAGCTT**GAGAACGAGAAAGATAATATCAACATGGACCCGGAGAAAAAGAACGAGAACGACCACAAGAAGGACGACAAGTACGACCACAAAGACGAGAACATGAAGAACGATCCAACGGCACAAAACAACCAGAACGAGGAGAACGACTTAACCAACATCGACGACGTCTTCGAGCGTAACAAG***GCG***AATGACTTCAACATCTCTCACGACAACATCACGAAAGACTTAAACCAGAAC | |
| IMC1g S_274/277_A  Re-codonised amino acids G_219_-R_283_ with KpnI site | GGAGAGCAGGACACCACAGTGGACACAATCACCCAGGAGAATTTTTGC**GGTACC**GTATCGTGCAACTTCTTGCCGAACTACCCGAATTTTAGTAAGATCGGGAATCCATTGTGTAAGGGTGGCCCAGAGAAGGAGAAGCGCTTCTCGTCAATTTCAATTTATAAG***GCA***AAAGAC***GCA***GGCTTTCCTTCGATTCGC | |
| NAB2 S_120_A  Re-codonised amino acids K_100_-M_297_ | AAACAGCACGACGAGTACTCGAAGTCAAATCGTTCGCGCGATAGCAAAAACCGTGTCAGC***GCA***GCTCACAATAAGAAGGGCGGTGAAGACGACTTCGACAAGCGTAACTTAAGCGACACCCGCCGCCGCAGTCGCTCATTGGCGTCCTCCAAATATAGTAACGAAGACATGTACTTCTCCAAAAACCGTAACAAACGTCGTCAAAAACGTGGACTTTCAATGCGTTCGGTTTCGTCATCTAGTAGTACCCGCAAAATCCATTATGATAAGAATAAGTCACGTAATAAACGCTCAGATCGCGGAAAGGACTCGGATATGTTCCGTTTGCGTGATAAATATCGTCGCTTGGGGAAATCTCACTCACAGTCATTTAGTCCCAGTCGTGTCATTTACGTGGAGAACGGGCAACGTAAGGAAAAAAAAAACGAGACTCACCACGACAACGTGTTAAATGTAAAGGACATCACGTACAAACGTATTAGTGACAGCGCGGATGAATATAATAATTCTGAGAAGAAAAATAAGGCGGTCCTTAAGCCAAACCCGCGTTTTGTAGGCGATAACCCAAATCCGTTCATGCAGCCTCCTACGATG | |
| Pf332 S_2346_A  Re-codonised amino acids V_2330_-D_2364_ with BamHI site | GTCGAGGTGGAGCGCTCGGCGACCGAGGACTTAGTAGAAGAGGAGGCC***GCT***GTGACCGAGAAGGTCAGCGTTCACGAA**GGATCC**ACAACCGAGCAGATTTTGGAC | |
| SEA1 S_280_A  Re-codonised amino acids E_257_-N_448_ with EcoRI site | GAGAACCAGAAGGATATCATCTATCTGAACAACCTGAACAATATCATGATGGACAAGTACAGCAACTGC***GCG***GACTCGCGCAAGAAAGAGTACTCTCACTTTAACTCCCAA**GAATTC**TCGTACGACAAGTACAGCATGAAGGATAGGATGTTCCTGAAGAACTTATACATGAAGCAGAACCGCCTGCGCGACAAGCGCGGCAAGTACCATAAGTTAGGAGACTACCAGAACATCGAGAATTACCGCAAGACAGGAGAGCACAGCTTCGACTGCATGAACATGTCCGACATCATGCACTCCAACAAGATGAGTCACGTTAACATAATGGACCATATGATCTACAAGGATAACAACAACATGAGTAAGCTAGTAGACACTATTAACTCACGCGAGAAAGACGTAAAGAACTATGATGACAATTTCGAGAGTTACAACAACTTCTTTAAAAACAATAACGACGAGCAGCATATTTGCTTAGAGTATGATGACACTTACAACTTAAAGGATACTGTAAAGAACATTATCGTGGAGGAGGAGCAGTGCGGTAAAGGAGTTGCTTGCATCTGCGACAAGAAT | |
| YOP1 S_28_A  Re-codonised amino acids R_15_-P_101_ | CGCCCAAACACCTCCTTGAACAGCCTGAAGCGTATCTCC***GCG***AATGTATTCGGAGAGAAGCTtAACAACTTGGACGTAAGCCGCGTTTTCAATAACATTGACGACTACGTCAAGAAGTACCCTTTCCTTAACAACATTGGAAAGAAGTTCGGAGTCAAACCCAGCTACATCATTGTCCCCTTCTCTGTGTTCTTATTCTTGTCATTAGTATTCGGGTGGGGAGCGGCTATCATCTGCAACGTTGTGGGGTTCGCATACCCA | |
| GAP45 re-codonised base pairs 50-615 (RR1) | ATATTGATGAACTTGCAGAGCGTGAAAATCTCAAAAAACAATCAGAAGAGATCATCGAGGAAAAACCAGAGGAAGTTGTTGAACAGGTCGAGGAGACCCATGAAGAACCGCTGGAGCAgGAgCAaGAATTGGACGAACAGAAAATTGAAGAAGAAGAAGAAGAACCGGAACAAGTTCCGAAGGAAGAAATCGATTACGCGACCCAAGAGAACAAAAGCTTCGAAGAAAAACATCTGGAGGATCTGGAGCGTTCAAACTCGGATATTTATTCGGAAAGCCAGAAGTTTGACAATGCCTCGGATAAACTGGAAACTGGTACCCAACTGACTCTGTCTACCGAAGCCACTGGGGCGGTCCAGCAGATTACTAAACTTTCGGAACCAGCCCACGAaGAATCAATTTACTTtACTTACaggAGCGTcACGCCCTGTGACATGAATAAACTCGATGAAACTGCCAAGGTTTTCAGCCGCCGTTGTGGTTGTGATCTGGGTGAACGCCATGACGAAAACGCGTGTAAGATTTGCCGCAAAATCGATCTTTCCGACACACCGCTCCTGAGCTGA | |
| GAP45 re-codonised base pairs 50-615, incorporating HA3 epitope tag and S_149_A & S_156_A mutations (RR2) | ATATCGACGAACTGGCGGAACGTGAGAACTTGAAAAAA**TACCCTTACGATGTTCCTGACTATGCGGGCTATCCCTATGACGTCCCGGACTATGCCATGGGCTACCCTTACGACGTTCCAGATTACGCT**CAATCTGAAGAGATCATTGAGGAGAAACCAGAGGAGGTCGTGGAACAGGTTGAGGAAACCCACGAAGAACCATTAGAGCAGGAGCAAGAGCTCGACGAGCAGAAAATTGAAGAGGAAGAGGAAGAACCGGAACAGGTCCCGAAAGAAGAAATCGATTACGCGACTCAGGAAAACAAGAGCTTTGAAGAAAAACATCTGGAGGACCTTGAACGCTCCAACTCTGATATTTACTCCGAGTCGCAGAAATTCGACAACGCCAGCGACAAACTGGAGACCGGAACCCAACTGACGCTCAGCACGGAAGCTACGGGAGCGGTTCAACAGATCACGAAGTTGTCGGAGCCGGCTCATGAGGAG***GCC***ATCTATTTCACCTATCGT***GCA***GTTACCCCGTGCGATATGAATAAACTGGACGAAACCGCAAAGGTCTTCTCGCGCCGTTGTGGCTGCGATCTGGGTGAACGTCATGACGAGAATGCCTGCAAAATCTGCCGCAAAATCGATCTGAGTGATACCCCTCTGTTATCGTAA | |
|  | | |
| **Table S2C: PCR primers used in the validation of the integration and, for *GAP45*, excision of phosphosite mutation constructs** | | |
| **PCR** | **Primer sequences** | **Upon modification** |
| *PF3D7_0414900* (*ARO*) base pairs 1-1808 | F: ATGGGAAATAATTGCTGTGCAGGAA  R: TTCGGGGATTTCATCTCCATCATTC | Introns 1-4 are removed, shortening PCR product to 1251 bp |
| *PF3D7_1251200* (Coronin) bp 993-2145 + 6 bp 3’UTR | F: TGATGGATTTGGTGGTGAAGACAAATG  R: CCCTTTTCATAATACCGTTGCTGTAC | HpaI site introduced into PCR product, gives 853 & 306 bp fragments |
| *PF3D7_1434500* (*DynR*) base pairs 19,972-21,718 | F: TCCTTTGACAAAATCAAGAAGAATTCT  R: CATCTTCCCATTTTTGATTATCTTGGCC | HindIII site introduced into PCR product, gives 886 & 861 bp fragments |
| *PF3D7_0525800* (*IMC1g*) base pairs 8-888 | F: CTACAAATAAGAATTTAGCTTGCTGC  R: GTAAAGATTTCTTTGGAACATTTGTGGAG | KpnI site introduced into PCR product, gives 700 & 189 bp fragments |
| *PF3D7_0623100* (*NAB2*) base pairs 348-2502 | F: GTAGACTGGTTAATGAGACTTATGGG  R: ATTAGAGACCTCTCCAACCGATTGC | Introns 2-6 are removed, shortening PCR product to 1314 bp |
| *PF3D7_1149000* (*Pf332*) base pairs 7140-8005 | F: CAGTTACTGACGAGGTCATAGAAGA  R: GTCTTCTGCAACTTCAGTGTTTGATCCTTC | BamHI site introduced into PCR product, gives 704 & 162 bp fragments |
| *PF3D7_1021800* (*SEA1*) base pairs 135-1940 | F: GAATGAAAACGATGGTATATGTGAA  R: TCACGTAGCTCATTACTAAGATCCA | EcoRI site introduced into PCR product, gives 1060 & 786 bp fragments |
| *PF3D7_0316700* (*YOP1*) 6bp 5’UTR + gene + 464bp 3’UTR | F: GACAAAATGAAAATGACCAAGTTGT  R: CATATATGCTATAGGGGTATGAACC | Intron is removed, shortening PCR product to 1135 bp |
| *PF3D7_1222700* (*GAP45*) | F: TGTTTAATACATACTGTGTAATCCTT  R: CCAAGATCACATCCACATCTTCTTGA | Locus PCR product size lengthens from 1116 bp to 2080 bp upon transgene integration, and shortens to 1417 bp upon RAP-induced gene excision |
