# Supplementary material for: Malaria Parasite Schizont Egress Antigen-1 Plays an Essential Role in Nuclear Segregation during Schizogony
Source: mBio. 2021 Mar 9;12(2):e03377-20. doi: 10.1128/mBio.03377-20 (PMC8092294; doi:10.1128/mBio.03377-20)
Supplement: TEXT S1 [file mBio.03377-20-s0001.docx]

**SUPPLEMENTARY METHODS**

**Generation of phosphosite mutations in 3D7 parasites**

Serine to alanine substitution mutations were generated by Cas9-mediated genome editing of *P. falciparum* 3D7 parasites. pDC2-based plasmids encoding sgRNAs targeting the sequences indicated in Table S2A were co-transfected with a linear repair construct recodonised region (Table S2B) flanked by homology arms of ~600 base pairs. Two clones for each genetic mutations were obtained by limiting dilution and validated by capillary sequencing and PCR based approaches described in Fig. S2 and Table S2C.

**Generation of the *GAP45lox_S_149/156_A* line**

The *GAP45lox_S_149/156_A* line was generated by very similar methods as those previously described for the tagging and conditional deletion of GAP45 (17). Briefly, the endogenous *GAP45* sequence was replaced using a Cas9-mediated genome editing to incorporate two differently-recodonised versions of base pairs 50-615 downstream of the endogenous 5’ 49 base pairs of the gene. Synthetic *SERA2* introns (described previously (18)) containing a *lox71* or *lox66* site were placed upstream of the first and second recodonised regions respectively. The second recodonised region included an HA3 tag within a low-complexity region as described previously (17,38) and mutations that would be translated into alanine instead of serine residues at amino acid positions 149 and 156. Clones were obtained by limiting dilution. Treatment of the clones with RAP resulted in genetic excision between the *lox* sites (Fig. S3A & B) and the replacement of the native C-terminus of the GAP45 protein with one containing an HA_3_ epitope tag (Fig. S3A, C & D) and alanine residues at positions 149 and 156. The *in vitro* growth of the resulting *GAP45lox_S_149/156_A* line was unaffected by treatment with RAP (Fig. S3E), indicating that phosphorylation of Ser_149_ and Ser_156_ is not essential for efficient merozoite egress and invasion.

**REFERENCES (SUPPLEMENTARY METHODS)**

17. Perrin AJ, Collins CR, Russell MRG, Collinson LM, Baker DA, Blackman MJ. The Actinomyosin Motor Drives Malaria Parasite Red Blood Cell Invasion but Not Egress. MBio. 2018.

18. Jones ML, Das S, Belda H, Collins CR, Blackman MJ, Treeck M. A versatile strategy for rapid conditional genome engineering using loxP sites in a small synthetic intron in *Plasmodium falciparum*. Sci Rep. 2016.

38. Ridzuan MAM, Moon RW, Knuepfer E, Black S, Holder AA, Green JL. Subcellular location, phosphorylation and assembly into the motor complex of GAP45 during *Plasmodium falciparum* schizont development. PLoS One. 2012.
